# YY1^lo^ NKT cells are dedicated IL-10 producers

**DOI:** 10.1038/s41598-020-60229-6

**Published:** 2020-03-03

**Authors:** Patrick W. Darcy, Lisa K. Denzin, Derek B. Sant’Angelo

**Affiliations:** 1 Graduate School of Biomedical Sciences, Rutgers Robert Wood Johnson Medical School, New Brunswick, NJ 08901 USA; 2 Department of Pediatrics, Rutgers Robert Wood Johnson Medical School, New Brunswick, NJ 08901 USA; 3 Child Health Institute of New Jersey, Rutgers Robert Wood Johnson Medical School, New Brunswick, NJ 08901 USA

**Keywords:** Interleukins, NKT cells, Tumour immunology

## Abstract

Co-expression of Yin Yang 1 (YY1) is required for the full function of the transcription factor, PLZF, which is essential for the development of natural killer T cell (NKT cell) effector functions. Discordant expression of YY1 and PLZF, therefore, might define NKT cell subsets with distinct effector functions. A subset of NKT cells was identified that expressed low levels of YY1. YY1^lo^ NKT cells were found in all tissues, had a mature phenotype and, distinct from other NKT cells, expressed almost no ThPOK or Tbet. When activated, YY1^lo^ NKT cells produced little IL-4 or IFN-γ. YY1^lo^ NKT cells were found to constitutively transcribe IL-10 mRNA and, accordingly, produced IL-10 upon primary activation. Finally, we find that tumor infiltrating NKT cells are highly enriched for the YY1^lo^ subset. Low YY1 expression, therefore, defines a previously unrecognized NKT cell subset that is committed to producing IL-10.

## Introduction

Invariant Natural Killer T cells (NKT cells) are a highly conserved population of αβ T cells associated with many types of immune responses^1^. Mouse NKT cells are defined by the expression of an invariant TCR α-chain (TCRVα14Jα18) that pairs with a limited repertoire of TCRVβ chains including TCRVβ8.2, TCRVβ7, and TCRVβ2^2^. This TCR complex recognizes glycolipid antigens presented in the context of CD1d^3^. Primary activation of NKT cells, either by the recognition of lipid antigens or inflammatory cytokines leads to rapid and copious cytokine production^4^. The potent production of this multitude of cytokines plays a major role in the ability of NKT cells to execute such diverse effector functions.

Differential expression of key transcription factors has been used to define NKT cell subpopulations. NKT1 cells, for example, express T-box 21 (Tbet) and are skewed towards the production of Th1 cytokines including IFN-γ, NKT2 cells, which express higher levels of GATA binding protein-3 (GATA3), produce IL-4 and retinoic acid receptor-related orphan nuclear receptor gamma (RORγt) expressing NKT17 cells, produce the cytokine IL-17^5,6^. Although these three subgroups are convenient for comparison of NKT cell responses and development, it has been shown that cytokine production by NKT cells is actually astonishingly diverse. In one particularly extensive study, nearly 20 different cytokines were detected post activation, including IFN-γ, IL-2, IL-3, IL-4, IL-5, IL-6, IL-9, IL-10, IL-13, IL-17, IL-21, GM-CSF, TNF, MIP-1α, RANTES, IL-12, and MCP-1^7^. The variety in the expression of these different cytokines suggests that there might actually be a multitude of distinct NKT cell effector subsets. It is not clear, however, if distinct cytokine expression patterns are defined during development or are a consequence of differentiation following activation. Interestingly, many of the gene loci for these cytokines are constitutively in an “open” configuration in resting NKT cells, resulting in a continuous basal level of mRNA transcription^8,9^. The one clear commonality between all NKT cell effector subsets is the requirement for the expression of the BTB-ZF transcription factor, PLZF^10,11^. In the absence of PLZF, NKT cells develop, but fail to acquire nearly all of their unique defining characteristics, including the capacity to rapidly produce cytokines upon primary activation. Importantly, the hallmark constitutive transcription of cytokine genes is dependent upon PLZF^12^.

Among the many cytokines made by NKT cells, the ability to produce IL-10 is of particular interest. IL-10 is a potent immunomodulator that, for example, impacts the growth of tumors and controls inflammatory bowel and autoimmune diseases^13^. Although many leukocytes can make IL-10, NKT cell specific IL-10 production has been associated with multiple immune responses. For example, control of homeostasis in the visceral adipose tissue is dependent on IL-10 from NKT cells^14^, as are some aspects of cardiac remodeling^15^. Autoimmune diseases ranging from alopecia areata^16^ to insulin-dependent diabetes mellitus^17^ are associated with IL-10 producing NKT cells^18,19^, as is the growth of intestinal polyps that can lead to cancer^20^.

The origin of IL-10 producing NKT cells (sometimes referred to as NKT10), however, is not clear^21,22^. Unlike the NKT1, NKT2, and NKT17 subpopulations^6^, a definitive transcription factor expression pattern that specifically identifies NKT10 cells has not been identified. Furthermore, the study of IL-10 producing NKT cells, to this point, has largely relied upon first activating NKT cells, followed by reactivation of the cells to detect IL-10^21,22^. Studies such as these have actually shown that all of the defined NKT cell effector subgroups can be induced to express IL-10^21^. One study suggests that pre-existing “natural NKT10” cells are present in the mouse^22^. This study, however, reports that these cells can only be detected 16 hours after *in vivo* activation with the strong agonist ligand, αGalCer. Therefore, the consensus is, that similar to conventional T cells, IL-10 production by NKT cells is a consequence of activation induced differentiation.

In this study, we find that a population of NKT cells, primed to secrete IL-10 following primary activation, is marked by low expression of the transcription factor Yin Yang-1 (YY1). A polycomb group protein, YY1 is thought to modulate chromatin architecture both by mediating the formation of promoter-enhancer loops and recruiting HDAC and HAT proteins to specific genetic loci^23,24^. Multiple biological processes have been shown to be dependent upon YY1, including oncogenisis^25^, hematopoeisis^26^ and heart development^27^ as well as several facets of T cell biology. During T cell development, YY1 is required for suppression of p53 mediated apoptosis of immature thymocytes^28^. In mature CD4 T cells, YY1 cooperates with lineage defining transcription factors such as FoxP3 in Tregs^29,30^ and Gata3 in Th2 cells^31^ to coordinate each effector lineages specific gene expression program. YY1 has also been found to play an important role during the commitment of CD8 T cells to effector lineages as opposed to the memory lineage^32^.

In a recent publication, we found that YY1 is required for NKT cell effector functions. YY1 deficient NKT cells failed to produce the burst of cytokines characteristic of primary NKT cell activation, despite expressing wild type levels of PLZF^33^. Therefore, YY1 co-expression in NKT cells is required for the function of PLZF. The control of YY1 by PLZF may be direct, since the transcription factors were shown to be in a complex. To further explore how PLZF and YY1 might cooperate to regulate effector functions in NKT cells, we examined expression of the two proteins. These studies identified a small population of NKT cells that expressed low levels of YY1 as compared to PLZF. In the thymus, YY1^lo^ NKT cells mostly had a mature, Stage 3, phenotype. Also, consistent with being mature cells, YY1^lo^ NKT cells were found in all examined tissues, including the spleen, liver and lungs. Despite the mature phenotype, YY1^lo^ cells expressed very low levels of both Tbet and ThPOK. Most interestingly, when activated, YY1^lo^ NKT cells produced little IL-4 and IFN-γ, but rather, produced IL-10. Finally, we find that YY1^lo^ NKT cells selectively accumulate in tumors. Thus, our data identify a subset of invariant NKT cells that is dedicated for producing IL-10.

## Results

### A population of NKT cells in the thymus expresses low levels of YY1

Nearly all of the most potent effector functions of NKT cells, including the exceptionally rapid response to activation, resulting in the production of a massive burst of cytokines, require expression of the transcription factor PLZF^11,12^. We recently showed, however, that many of the functions of PLZF are dependent upon expression of YY1, a transcription factor itself, that we find is in association with PLZF^33^. Of particular interest, YY1 deficient NKT cells, we found, express wild type levels of PLZF, but do not produce cytokines following primary activation^33^.

**Figure 1:**
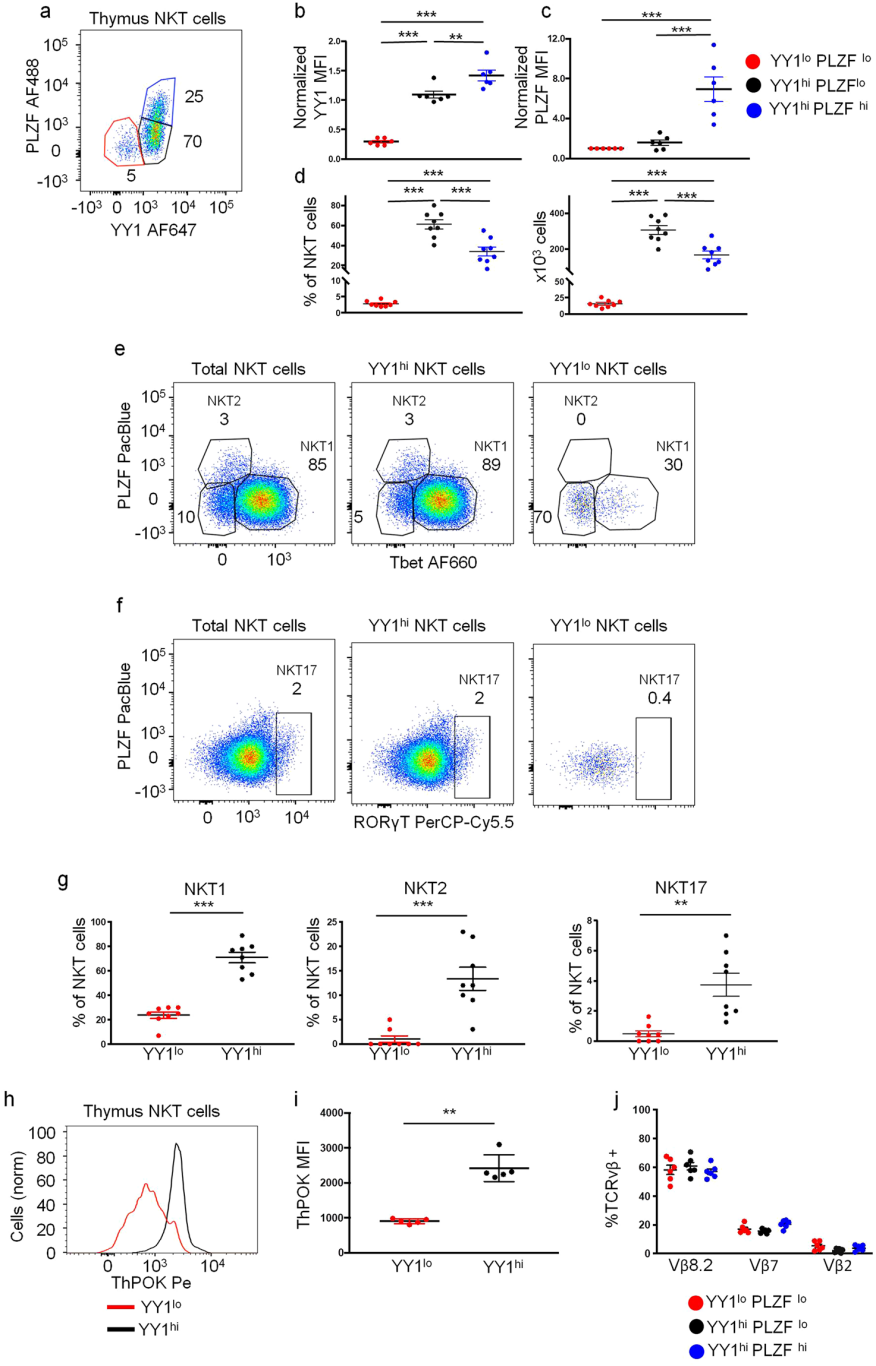
A population of NKT cells in the thymus expresses low levels of several transcription factors known to regulate NKT cell effector functions. Thymuses were collected from C57BL/6J (WT) mice and analyzed by FACS with the indicated antibodies. (**a**–**d**) YY1 and PLZF expression levels were determined in WT NKT cells (MHCII^−^, CD3^+^, CD1d tet^+^, CD24^−^). (**a**) NKT cells segregated into three populations on the basis of YY1 and PLZF expression: YY1^lo^ PLZF^lo^ (red), YY1^hi^ PLZF^lo^ (black), and YY1^hi^ PLZF^hi^ (blue). (**b**) Mean fluorescence intensity (MFI) of YY1 and (**C**) PLZF expression in the three subsets and (**d**) the percentage and absolute cell number of NKT cells in each of the three subpopulations. YY1 MFI measurements were normalized to expression in CD4 single positive thymocytes stained in the same experiment. PLZF MFI measurements were normalized to YY1^lo^ NKT cells stained in the same experiment. (**e**–**g**) Intracellular staining for (**e**) Tbet and PLZF or (**f**) RORγt and PLZF in all thymic NKT cells (left), YY1^hi^ NKT cells (middle), and YY1^lo^ NKT cells (right). (**g**) Distribution of YY1^lo^ and YY1^hi^ NKT cells in the NKT1, NKT2, and NKT17 subpopulations as defined in (**e**,**f**). (**h**,**i**) ThPOK expression was compared between YY1^lo^ and YY1^hi^ NKT cells by FACs. Representative histogram in (**h**) and cumulative data in (**i**). (**j**) TCRVβ usage, measured by FACs, was compared between YY1^lo^, YY1^hi^ PLZF^lo^, and YY1^hi^ PLZF^hi^ NKT cells. FACS plots show representative results from 3–5 independent experiments. (**b**,**c**,**f**,**g**,**i**,**j**) show compiled data from at least 3 independent experiments. For (**i**) N = 5, (**b**–**d**,**j**) N = 6, (**g**) N = 7. Each dot depicts one mouse. The horizontal lines indicate the mean (±s.e.m.). *P < 0.05, **P < 0.01 ***P < 0.001 determined by One-Way Anova (**b**,**c**,**d**,**j**) or Mann-Whitney U Test (**g**,**i**).

NKT cell subpopulations with distinct cytokine production profiles express different levels of PLZF^6^. Since YY1 is required for PLZF function, we reasoned that noncoordinate expression of the two transcription factors might correlate with distinct functionality. Expression levels of the two transcription factors were quantified in NKT cells from the thymuses from 8 week old C57BL/6J mice. By comparison of PLZF to YY1 expression levels, three subsets of NKT cells were identified (Fig. 1a). YY1 expression was highest in NKT cells that also had the highest level of PLZF (Fig. 1a–c). These YY1^hi^PLZF^hi^ NKT cells accounted for roughly one third of the total NKT cell population (Fig. 1d). The largest subpopulation, making up more than half of the NKT cells (Fig. 1d), expressed somewhat lower levels of YY1 and, also, lower levels of PLZF. Therefore, in the bulk of NKT cells, levels of PLZF and YY1 mimicked each other. However, the smallest of these subpopulations expressed much lower levels of YY1, but only slightly lower levels of PLZF (Fig. 1a–c). The frequency and cell numbers of the three populations, including the small, YY1^lo^PLZF^lo^ population (ranging from 3–5% of total NKT cells) were consistent from mouse to mouse (Fig. 1d). Differential YY1 expression was also observed in other thymic T cell populations (Supp. Figure 1). Therefore, although YY1 is ubiquitously expressed in all NKT cells, the level of expression varied among these newly defined subsets. Furthermore, in one population, the expression of the two factors was found to be discordant.

NKT cells require Tbet to fully acquire their characteristic effector/memory cell surface phenotype and to express the cytokine IFN-γ^34^. RORγt expression is necessary for mature NKT cells to produce the inflammatory cytokine IL-17^35,36^. Mature NKT cells can be segregated into effector subpopulations including NKT1 (Tbet^hi^ PLZF^lo^), NKT2 (Tbet^lo^ PLZF^hi^) or NKT17 (RORγt^+^)^6^. The majority of YY1^hi^ NKT cells (for simplicity, the two YY1^hi^ populations are combined here) were found to be Tbet^hi^ PLZF^lo^ similar to NKT1 cells. (Fig. 1e,g) A small percentage were Tbet^lo^ PLZF^hi^ consistent with the NKT2 phenotype (Fig. 1e,g). In contrast, YY1^lo^ NKT cells were mostly Tbet^lo^ PLZF^lo^ (Fig. 1e,g) and, therefore, based on transcription factor expression were distinct from the well-described NKT cell effector subpopulations. ThPOK has also been shown to be essential for NKT cell effector functions^37,38^. Most notably, ThPOK deficient NKT cells do not produce IL-4 following primary activation. Interestingly, NKT cells in ThPOK KO mice are skewed towards the NKT17 lineage. YY1^lo^ NKT cells expressed substantially lower levels of ThPOK as compared to YY1^hi^ NKT cells (Fig. 1h and Supp Fig. 1). The YY1^lo^ NKT cells, however, did not express RORγt (Fig. 1f,g).

TCR Vβ gene segment usage has been shown to correlate with the effector lineage adopted by developing NKT cells. We determined if YY1^lo^ NKT cells had differential TCR Vβ usage that might be associated with their unique characteristics^39^. 60% of YY1^lo^ NKT cells expressed TCR Vβ8.2, 15% of YY1^lo^ NKT cells expressed TCR Vβ7, and 5% of YY1^lo^ NKT cells expressed TCR Vβ2 (Fig. 1i). These TCR Vβ usage frequencies were similar to YY1^hi^PLZF^lo^ NKT cells and YY1^hi^PLZF^hi^ NKT cells, suggesting that differential TCRVβ usage did not impact the unique transcription factor expression pattern of the YY1^lo^ NKT cells.

### YY1^lo^ NKT cells in the thymus are not at an immature or transient population

Based on the expression of several key transcription factors, our data suggested that the YY1^lo^ NKT cells might either be an immature or a functionally distinct population of NKT cells. As NKT cells develop, they upregulate cell surface markers associated with effector/memory T cells including CD44, CD69, and CXCR3 as well as markers typically found on NK cells such as NK1.1^40,41^. Since differential expression of these cell surface markers on NKT cells is associated with maturation, we analyzed expression on the YY1^lo^ subset.

**Figure 2:**
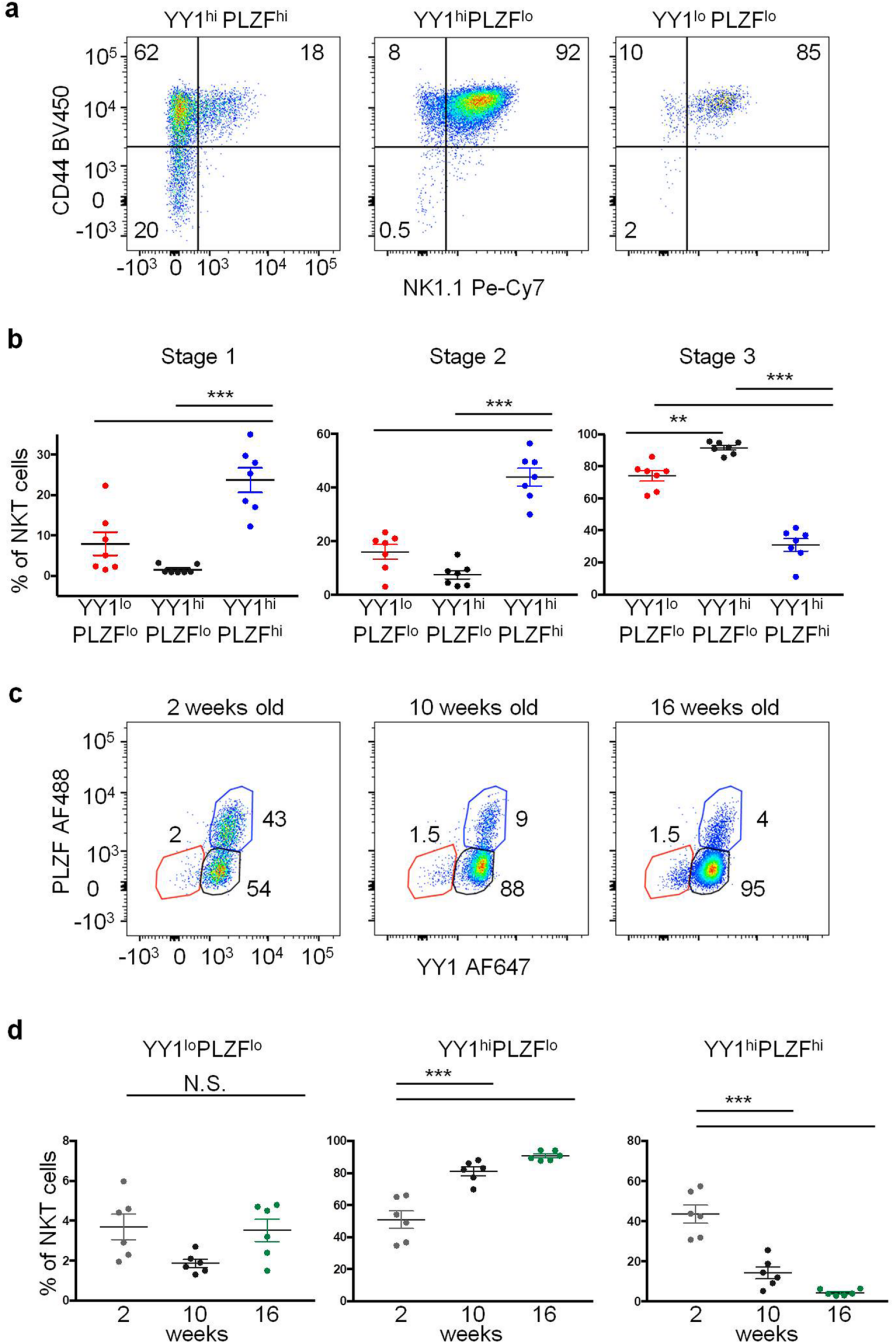
YY1^lo^ NKT cells are not an immature or transient population. Thymuses were collected from C57BL/6J (WT) mice and analyzed by FACS with the indicated antibodies. (**a,b**) NKT cells (MHCII^−^, CD3^+^ CD1dtet^+^ CD24^−^) were stained for the transcription factors PLZF and YY1, as well as, the cell surface markers CD44 and NK1.1. Expression of CD44 and NK1.1 was compared between the YY1/PLZF subpopulations defined in (Fig. 1a). Representative FACs plots are shown in (**a**) and cumulative data concerning the distribution of NKT cells in different stages are plotted in (**b**). The NKT cell stages were defined as: Stage 1 (CD44^−^ NK1.1^−^), Stage 2 (CD44^+^ NK1.1^−^), and Stage 3 (CD44^+^ NK1.1^+^). (**c,d**) Thymus NKT cells isolated from 2 week old, 10 week old, and 16 week old WT mice were stained for YY1 and PLZF. (**c**) Representative FACs plots depicting YY1 and PLZF expression are shown. (**d**) Cumulative data comparing the frequency of each YY1/PLZF defined NKT cell subpopulation between mice of different ages. FACS plots show typical results from indicated specimen and graphs show compiled data from 7 (**b**) or 6 (**d**) mice, examined in 3 or more independent experiments. The horizontal lines indicate the mean (±s.e.m.). ***P < 0.001 determined by One Way Anova (**b**,**d**).

Thymic NKT cells from 8 week old C57BL/6J mice were stained for the surface markers CD44 and NK1.1 and the transcription factors YY1 and PLZF. NKT cells from all three YY1/PLZF subsets as defined in (Fig. 1a) were found to be distributed among all three of the described stages of development (Fig. 2a,b). The YY1^hi^ PLZF^hi^ population was mostly in Stage 1 (CD44^−^NK1.1^−^) or Stage 2 (CD44 + NK1.1^−^), with some cells progressing to Stage 3 (CD44 + NK1.1^+^). The majority of YY1^hi^ PLZF^lo^ cells, were concentrated in Stage 3, consistent with Tbet^+^ NKT1 type cells. Interestingly, despite little Tbet expression, the YY1^lo^ NKT cells largely had a Stage 3 phenotype (Fig. 2a,b). The expression of CD44 and NK1.1 strongly suggested that YY1^lo^ NKT cells were a mature population of NKT cells.

YY1 and PLZF expression was profiled in thymic NKT cells isolated from 2 week, 10 week and 16 week old C57BL/6J mice. An increase in the frequency of YY1^lo^ NKT cells would imply that this was an induced phenotype. The frequency of YY1^lo^ NKT cells, however, was consistent between mice of different ages (Fig. 2c,d). In contrast, the frequency of YY1^hi^PLZF^hi^ NKT cells and YY1^hi^PLZF^lo^ NKT cells changed substantially as mice aged (Fig. 2c,d). YY1^hi^PLZF^hi^ NKT cells were overrepresented in younger mice, whereas YY1^hi^PLZF^lo^ NKT cells were found at a significantly higher frequency in older mice, consistent with previous results^12^. The stable representation of YY1^lo^ NKT cells in both young and old mice implied that this subset is not a derivative of the other populations.

### YY1^lo^ NKT cells are found in secondary lymphoid and non-lymphoid tissues

**Figure 3:**
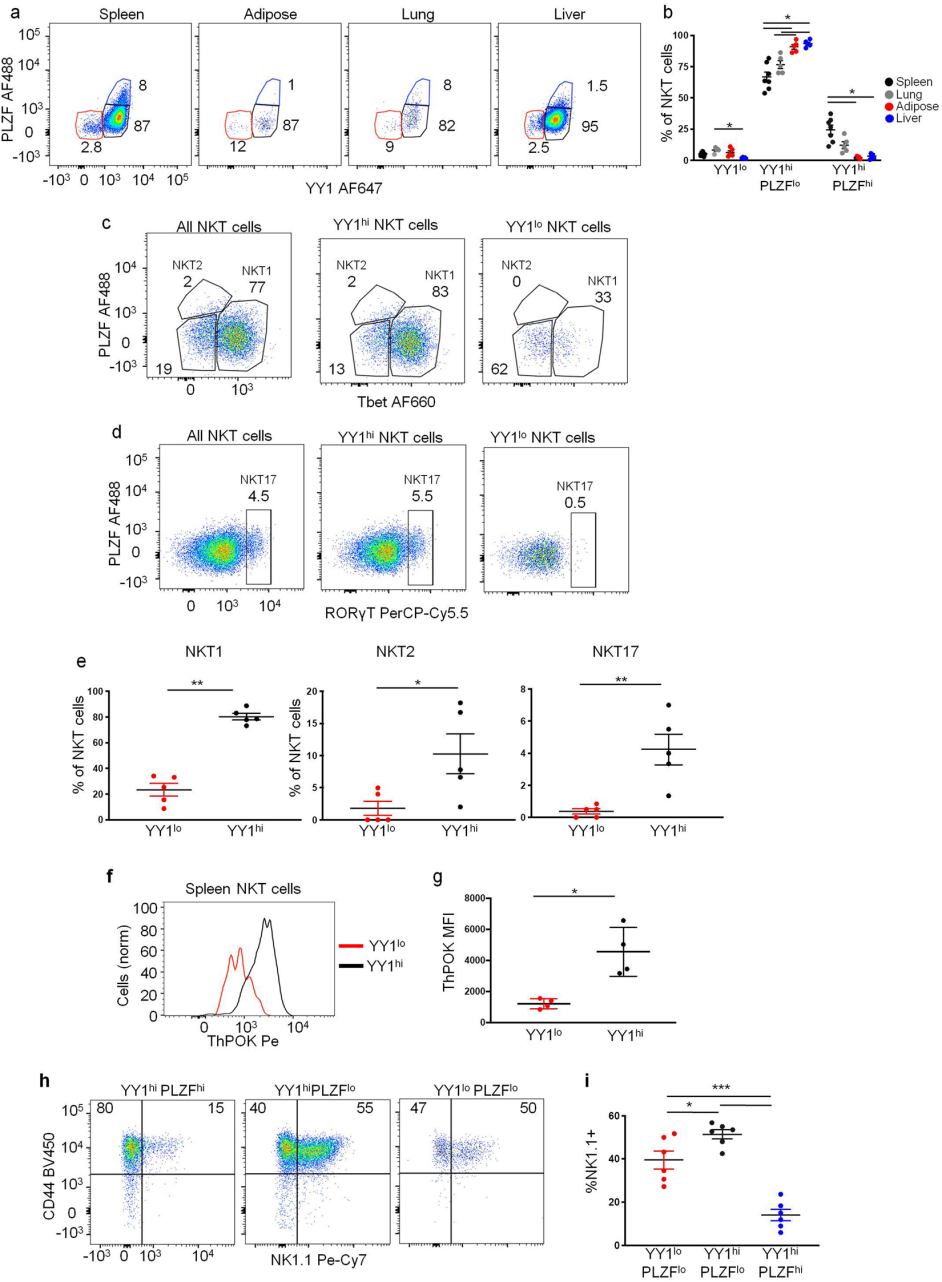
YY1^lo^ NKT cells are found in lymphoid and nonlymphoid tissues and share a common transcription factor expression pattern. Lymphocytes were isolated from the spleen, visceral adipose, lung, and liver of 12 week old, C57BL/6J (WT) mice and analyzed by FACS with the indicated antibodies. Expression of PLZF and YY1 in the NKT cells (CD45.2^+^, MHCII^−^, CD3^+^ CD1dtet^+^ CD24^−^) was determined. (**a**) Representative FACs plots and (**b**) cumulative data comparing the frequency of the YY1/PLZF subpopulations between tissues (**c–e**). Intracellular staining for (**c**) Tbet and PLZF or (**d**) RORγt and PLZF in total spleen NKT cells (left), YY1^hi^ NKT cells (middle), and YY1^lo^ NKT cells (right). (**e**) Distribution of spleen YY1^lo^ and YY1^hi^ NKT cells in the NKT1, NKT2, and NKT17 subpopulations as defined in (**c,d**). (**f,g**) ThPOK expression was compared between spleen YY1^lo^ and YY1^hi^ NKT cells by FACs. A representative histogram is shown in (**f**) and cumulative data are summarized in (**g**). (**h**,**i**)Expression of CD44 and NK1.1 was compared between the YY1/PLZF subpopulations. Data in (**a**,**c**,**d**,**f**,**h**) are representative of at least 4 independent experiments. Each dot in (**b**,**e,g,i**) represents an individual mouse, for (**b**) N ≥ 5 for each tissue and for (**e**,**g**) N = 4, for (**i**) N = 6. The horizontal lines indicate the mean (±s.e.m.). *P < 0.05 **P < 0.01, ***P < 0.001 determined by one-way ANOVA (**b,i**) or Mann-Whtiney U Test (**e**,**g**).

The abundance of different NKT cell effector subpopulations differs between tissues. 90% of the NKT cells found in the liver belong to the NKT1 subpopulation, while the majority of NKT cells in the lung are NKT17 cells and the adipose tissue is enriched for IL-10 producing NKT cells^42^. Given the variability in representation of NKT cell effector subpopulations in different tissues, we reasoned that the percent of YY1^lo^ NKT cells might fluctuate between tissues. NKT cells were isolated from the spleen, visceral adipose tissue, lung, and liver of 8 week old C57BL/6 mice. The expression of YY1 and PLZF in these NKT cells was measured by flow cytometry. All three YY1/PLZF based subpopulations, as defined in (Fig. 1a), were identified in all tissues (Fig. 3a). The majority of NKT cells in each tissue were YY1^hi^PLZF^lo^ (Fig. 3a,b). Approximately 3%-10% of NKT cells in each tissue belonged to the YY1^lo^PLZF^lo^ subpopulation (Fig. 3a,b), indicating that the relative abundance of YY1^lo^ NKT cells was not highly variable between tissues.

The frequency of NKT cell effector subpopulations also differs between mouse strains. NKT2 cells, for example, are found at a higher frequency in BALB/cJ mice as compared to C57BL/6J mice, whereas NKT1 cells are more abundant in C57BL/6J mice^6^. The frequency of YY1^lo^ NKT cells in the thymuses and spleens of BALB/cJ mice and C57BL/6J mice was, however, indistinguishable (Supplemental Fig. 2).

Similar to what was found for thymic NKT cells, few splenic YY1^lo^ NKT cells expressed high levels of Tbet (Fig. 3c) and they also did not express RORγt (Fig. 3d). Therefore, YY1^lo^ spleen NKT cells also appeared to be distinct from NKT1 cells, NKT2 cells, or NKT17 cells (Fig. 3e), which was in contrast to YY1^hi^ NKT cells (Fig. 3c–e). In addition, the YY1^lo^ NKT cells in the spleen expressed low levels of ThPOK (Fig. 3f,g). These data demonstrate that, similar to the thymus, YY1^lo^ NKT cells in the spleen have a distinct transcription factor expression pattern. The majority of the YY1^lo^ NKT cells in the spleen expressed NK1.1 (Fig. 3h,j) suggesting that some YY1^lo^ NKT cells were recent thymic emigrants, while others had been in the periphery for an extended period.

### YY1^lo^ NKT cells produce little IL-4 and IFN-γ following activation

**Figure 4:**
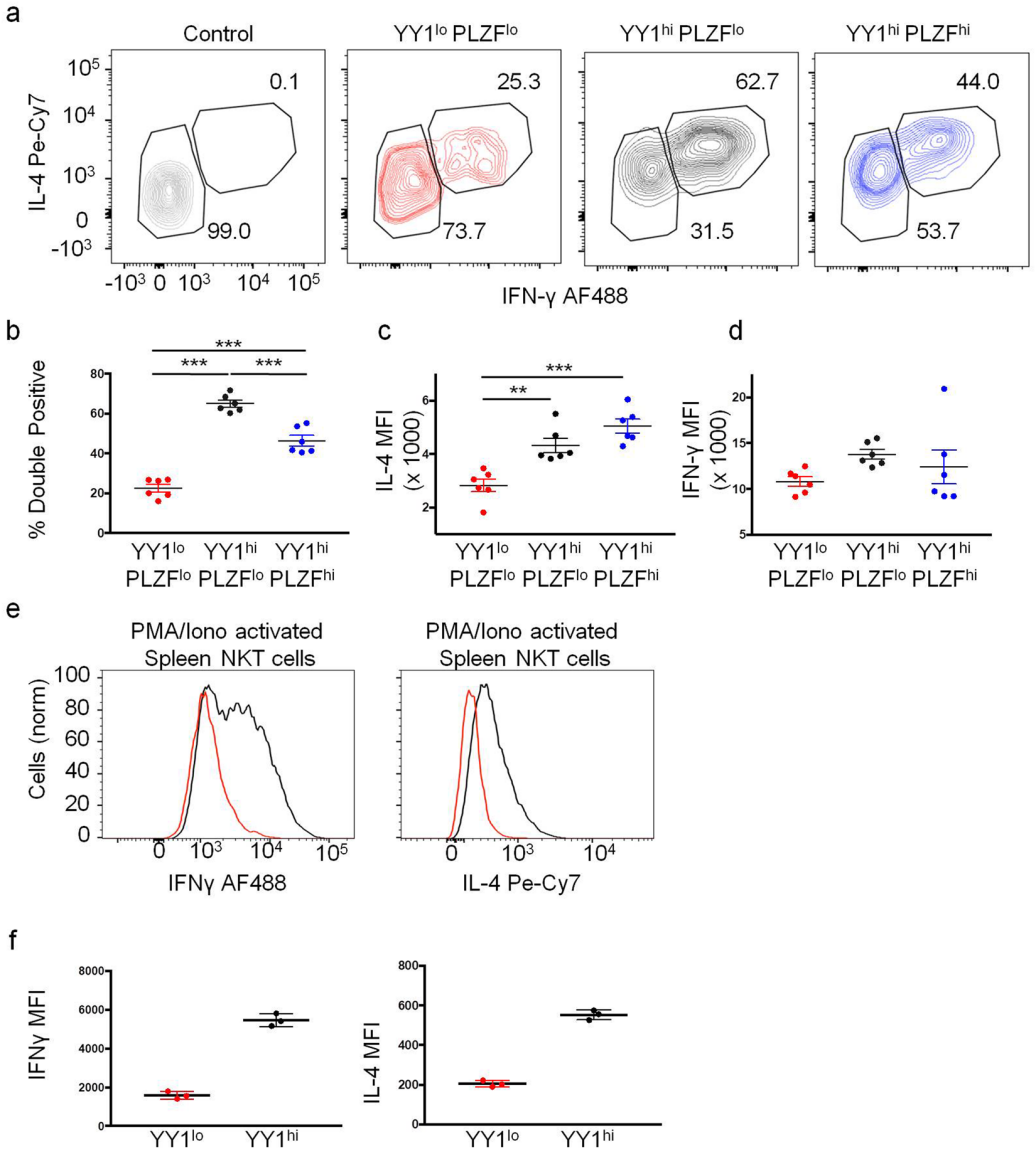
YY1^lo^ NKT cells produce less IL-4 and IFN-γ as compared to YY1^hi^ NKT cells. (**a**–**d)** C57BL/6J (WT) mice were injected I.V. with either α-GalCer (10*ug*) suspended in saline or saline only (control). 90 minutes after injection, splenic NKT cells were harvested and stained for IL-4 and IFN-γ. (**a**) Representative FACs plot for IL-4 and IFN-γ expression in each WT activated NKT cell (MHCII^−^, CD3^+^ CD1dtet^+^ CD24^−^) subset: YY1^lo^ PLZF^lo^, YY1^hi^ PLZF^lo^, and YY1^hi^ PLZF^hi^. (**b**) Cumulative data from 6 mice, depicting the % of IL-4^+^ and IFN-γ ^+^ double positive cells. The IL-4 MFI of IL-4^+^ cells (**d**) and IFN-γ MFI of IFN-γ^+^ cells (**d**) from each NKT cell subset is plotted. The IL-4 MFI and IFN-γ MFI of each experimental sample was normalized to the IL-4 MFI and IFN-γ MFI of control, unactivated NKT cells stained in the same experiment. (**e**–**f**) NKT cells were isolated from the spleens of WT mice and activated *in vitro* for 5 hours by culture with PMA (50 ng/mL) and Ionomycin (500n/mL). Activated NKT cells were stained for IL-4, IFN-γ, and YY1. (**e**) Representative histograms and (**f**) and aggregate data compare the production of IFN-γ (left) and IL-4 (right) between YY1^lo^ and YY1^hi^ NKT cells following *in vitro* activation. Data are representative of 4 (**a**–**d**) or 3 (**e**–**f**) independent experiments. Each dot represents one mouse, N = 6 for (**b–d**), N = 3 for (**f**). The horizontal lines indicate the mean (±s.e.m.). *P < 0.05 **P < 0.01, ***P < 0.001 determined by one-way ANOVA (**b**–**d**).

Low expression of Tbet, PLZF, and ThPOK suggested that YY1^lo^ NKT cells might have distinct effector functions as compared to other NKT cells. To test this, NKT cells were activated *in vivo* by intravenous (IV) injection of mice with 10 µg of the NKT cell specific agonist ligand, α-galactosylceramide (α-GalCer). 90 minutes after injection, splenocytes were harvested and stained for the transcription factors PLZF and YY1, as well as the cytokines IL-4 and IFN-γ. The percentage of YY1^hi^ NKT cells that produced IL-4 and IFN-γ (both the PLZF^hi^ and PLZF^lo^ subsets) was consistently 2–3 fold higher than the YY1^lo^ NKT cells (Fig. 4a,b). Furthermore, the amount of IL-4 produced by the responding YY1^lo^ cells, as measured by mean fluorescence intensity (MFI), was less than half that of the YY1^hi^ cells (Fig. 4c). The MFI of IFN-γ response was not statistically different (Fig. 4d). The difference in ability to produce IL-4 and IFN-γ following primary activation was even more exaggerated following activation with PMA plus ionomycin *in vitro*. These experiments resulted in almost no IL-4 and IFN-γ production from YY1^lo^ NKT cells as compared to YY1^hi^ NKT cells **(**Fig. 4e,f**)**. YY1^lo^ NKT cells also failed to produce IL-17a following *in vivo* activation with α-GalCer (Supp Fig. 3a).

### YY1^lo^ NKT cells include dedicated IL-10 producing cells

**Figure 5:**
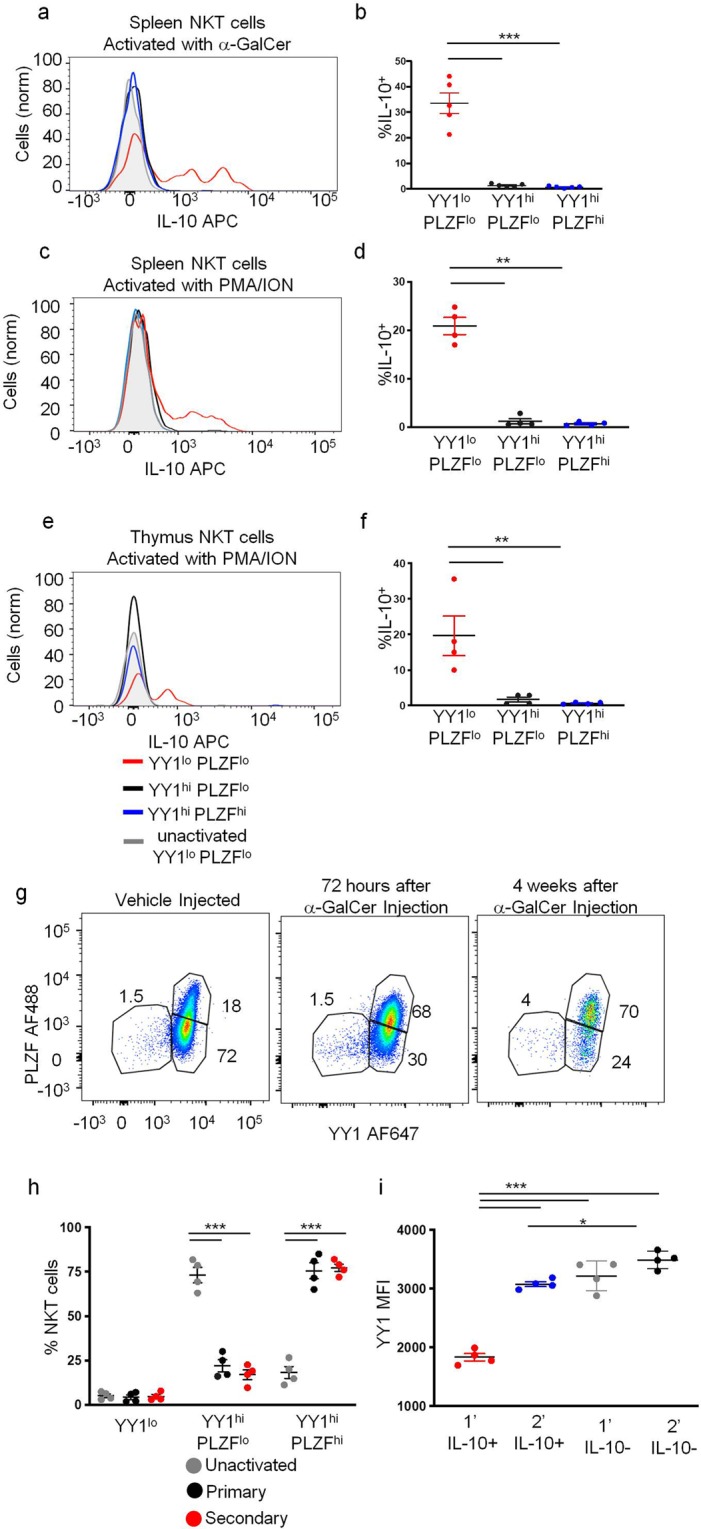
YY1^lo^ NKT cells produce IL-10 following primary activation. (**a**–**c**) C57BL/6J (WT) mice were injected I.V. with α-GalCer (10*ug*) suspended in saline. 90 minutes after injection, spleens were harvested and NKT cells (MHCII^−^, CD3^+^ CD1dtet^+^ CD24^−^) were enriched for by automacs. A cell surface capture assay was used to determine IL-10 production by YY1^lo^ PLZF^lo^, YY1^hi^ PLZF^lo^, and YY1^hi^ PLZF^hi^ NKT cells. (**a**) Representative histogram depicting IL-10 production by each subset. (**b**) Cumulative data from 5 mice, depicting the % of IL-10^+^ cells. (**c**–**f**) NKT cells were isolated from the spleens (**c,d**) or thymuses (**e,f**) of WT mice and activated *in vitro* for 3 hours by culture with PMA (50 ng/mL) and Ionomycin (500n/mL). NKT cell IL-10 production was measured by a cell surface capture assay. Representative histograms (**c,e**) and aggregate data (**d, f**), compare the production of IL-10 between YY1^lo^ PLZF^lo^, YY1^hi^ PLZF^lo^, and YY1^hi^ PLZF^hi^ NKT cells following *in vitro* activation. (**g**–**h**) C57Bl/6 (WT) mice were IV injected with 10 *u*g of α-GalCer or vehicle. Mice were sacrificed 72 hours or four weeks after injection. Spleen NKT cells (MHCII^−^, CD3^+^ CD1dtet^+^ CD24^−^) YY1 and PLZF expression were measured by FACs. (**g**) Representative FACS plots and (**h**) cumulative data. (**i**) C57Bl/6 (WT) mice were IV injected with 10 *u*g of α-GalCer or vehicle. 4 weeks later, NKT cells were enriched from spleens, activated *in vitro* with PMA/Ion, and IL-10 production was measured using a cell surface capture assay. Graph shows YY1 MFI and whether or not the cells produced IL-10. Data in histograms are representative of at least 3 independent experiments representing 3 or more biological replicates. For (b) N = 5, (d) N = 3, (**f,h,i**) N = 4. The horizontal lines indicate the mean (±s.e.m.). *P < 0.05determined by One-Way Anova (**b,d,f,h,i**).

NKT cells express many cytokines in addition to IL-4 and IFN-γ, including IL-10. To determine the capacity of YY1^lo^ NKT cells to produce IL-10, mice were injected IV with α-GalCer. 90 minutes after injection, mice were scarified and total spleen NKT cells were purified and a cell surface capture assay was used to detect IL-10 producing NKT cells. A substantial percentage of YY1^lo^ NKT cells were found to produce IL-10, while none was detected from YY1^hi^ NKT cells (Fig. 5a,b). Purified spleen NKT cells activated *in vitro* with PMA/ionomycin for 3 hours showed similar results (Fig. 5c,d). Interestingly, thymic NKT cells isolated and activated *in vitro* with PMA/ionomycin were also found to secrete IL-10 (Fig. 5e,f). Therefore, low expression of YY1 marks a population of NKT cells primed to secrete IL-10 following primary activation. Importantly, this subset exists in the thymus, suggesting it is not an induced phenotype caused by antigen activation.

Primary activation induces the differentiation of NKT cells such that upon secondary activation a large percentage produce a wide range of cytokines, including IL-10^21^. We tested the possibility that this increase in the frequency of IL-10 producing NKT cells following secondary activation was the consequence of an expansion of YY1^lo^ NKT cells following primary activation. We injected mice with 10 *u*g of α-GalCer and analyzed YY1 expression levels in NKT cells either 72 hours and or 4 weeks later. The frequency of YY1^lo^ NKT cells remained similar to vehicle injected mice at both time points (Fig. 5g,h**)**. Treatment with α-GalCer did, however, result in a substantial increase in the frequency of YY1^hi^PLZF^hi^ NKT cells and a corresponding decrease in the frequency of YY1^hi^PLZF^lo^ NKT cells in the spleen at both time points measured (Fig. 5g,h).

Next, we isolated NKT cells from the spleens of C57Bl/6J mice 1 month after IV injection of α-GalCer. These cells were reactivated *in vitro* with PMA and ionomycin for 3 hours. Data show that in addition to YY1^lo^ cells producing IL-10, prior activation with α-GalCer also enables some YY1^hi^ cells to produce IL-10 (Fig. 5i). Therefore, while YY1^lo^ NKT cells are committed IL-10 producing NKT cells, activation induced differentiation of YY1^hi^ NKT cells into IL-10 producing cells also occurs.

**Figure 6:**
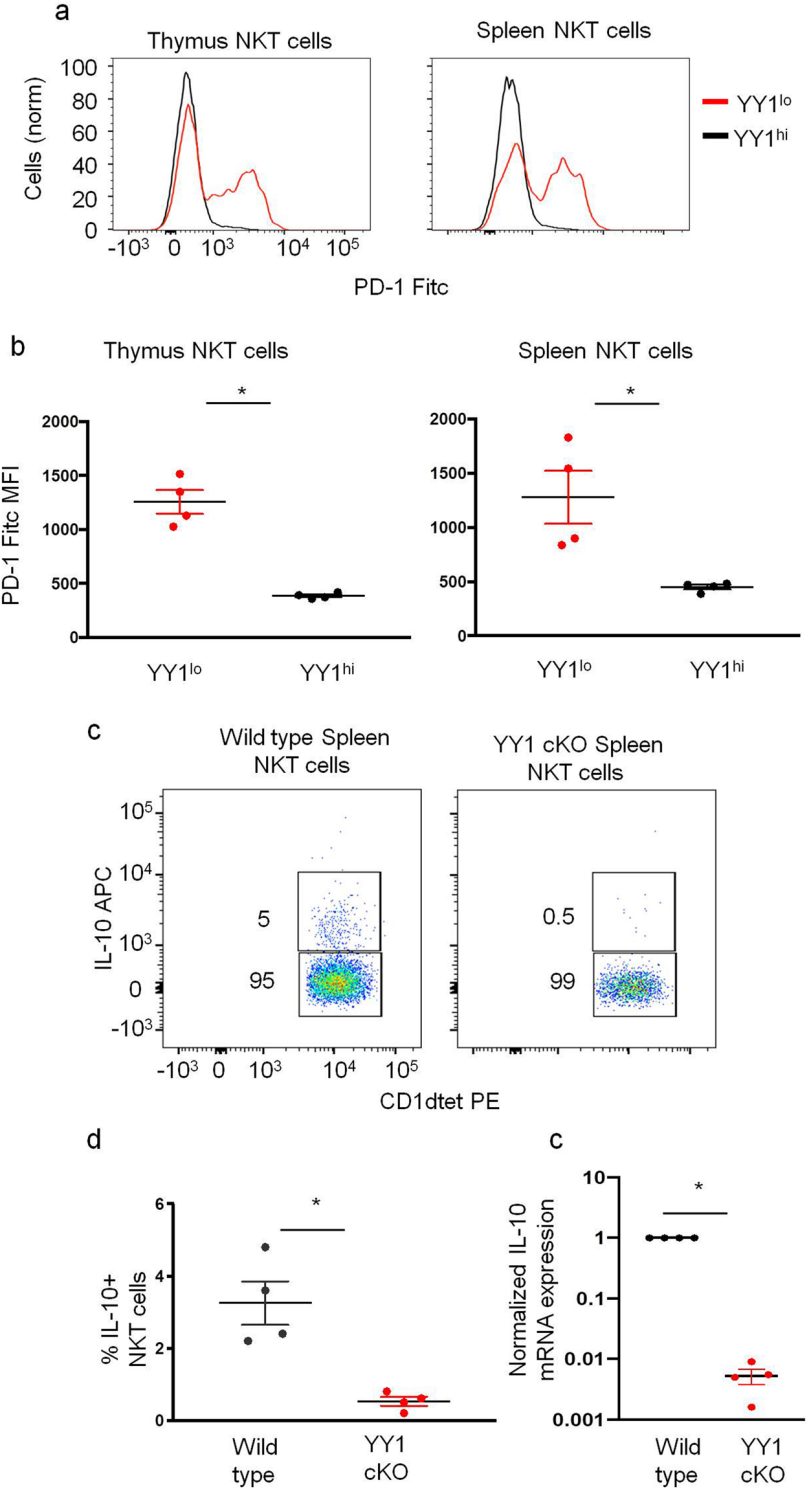
YY1 deficient NKT cells do not produce IL-10. (**a**,**b**) NKT cells from the thymus and spleen of C57BL/6J (WT) mice were stained for PD-1. Representative histogram in (**a**) and cumulative data from 4 mice in (**b**). (**c**–**e**) C57Bl/6 (WT) and PLZF-Cre YY1 flx.flx (YY1 cKO) mice were IV injected with 10 *u*g of α-GalCer. 90 minutes after injection, mice were scarified, NKT cells were enriched from spleens, and an IL-10 cell surface capture assay was used to measure NKT cell IL-10 production. (**c**) Representative FACs plot and (**d**) cumulative data. (**e**) NKT cells were FACs sorted from WT and YY1 cKO mice, RNA was isolated from sorted cells, and reverse transcribed into cDNA. qPCR measured β2 M and IL-10 expression in the sorted NKT cell populations. Data are from 4 or more independent experiments representing 4 biological replicates. The horizontal lines indicate the mean (±s.e.m.). *P < 0.05, ***P < 0.001, determined Mann Whitney U-Test (**b,d,e**).

Having demonstrated YY1^lo^ NKT cells to be dedicated IL-10 producers, we next determined the expression of transcription factors and cell surface markers associated with other IL-10 secreting NKT cell populations, namely arNKT cells and NKT10 cells, in YY1^lo^ NKT cells. In accordance with previous studies, we found that no NKT cells, including YY1^lo^ NKT cells, expressed the T_reg_ lineage defining transcription factor FoxP3 (Supplemental Fig. 3b). Like arNKT cells and NKT10 cells, YY1^lo^ NKT cells isolated from both the thymus and spleen were found to express high levels of PD-1 (Fig. 6a,b). Unlike NKT10 cells, YY1^lo^ NKT cells were found to express low levels of NRP1, CD49d and ICOS (Supplemental Fig. 3c). Differential expression of these three surface markers potentially can be attributed to differences in activation state between YY1^lo^ NKT cells, which were freshly isolated from naive mice and NKT10 cells, which were activated *in vitro* for four hours with PMA/Ionomycin.

### YY1 deficient NKT cells lose the capacity to rapidly produce IL-10

Since low YY1 expression marked an NKT cell population primed to rapidly produce IL-10, we tested the possibility that YY1 deficient NKT cells would have an enhanced capacity to produce IL-10. YY1 deficient NKT cells isolated from PLZF-cre YY1 flox/flox mice (YY1 cKO mice), however, produced little or no IL-10 following *in vitro* activation with PMA/ionomycin (Fig. 6c,d). To determine if YY1 deficient NKT cells failed to rapidly produce IL-10 as a consequence of losing their preformed IL-10 mRNA, mRNA was isolated from sorted YY1 cKO NKT cells, and qPCR experiments were performed. While preformed IL-10 message was detected in wildtype NKT cells, substantially less was detected in YY1 deficient NKT cells (Fig. 6e). YY1, therefore, like for other cytokines^33^, is required for rapid production of IL-10 in NKT cells, likely by opening the gene locus.

### YY1^lo^ NKT cell are enriched in tumors

**Figure 7:**
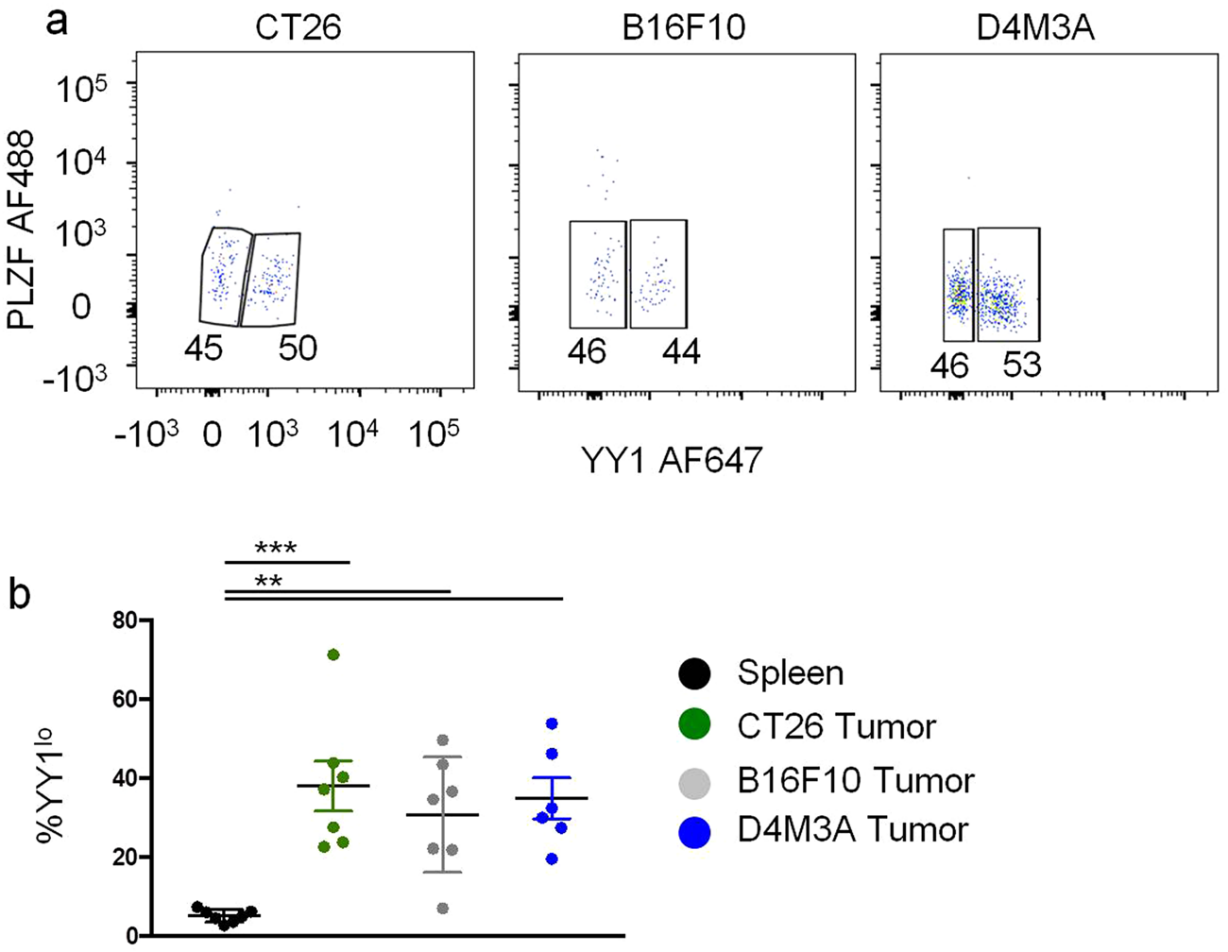
Tumors are enriched for YY1lo NKT cells. 5×10^5^ tumor cells were injected intradermally into mice. When tumors reached an area between 8×8 millimeters and 10×10 millimeters mice were sacrificed, tumors were harvested, and leukocytes were isolated from tumors. CT26 colon cancer cells were injected into BALB/CJ mice, while B16F10 melanoma cells and D4M3A melanoma cells were injected into C57BL/6J mice. (**a,b**). YY1 and PLZF expression in NKT cells (CD45.2^+^, CD19^−^, MHCII^−^, CD3^+^, CD1dtet^+^) isolated from tumors was determined by flow cytometry. A representative FACs plot for each tumor type is shown in (**a**) and cumulative data are shown in (**b**). Data are from 2 or more independent experiments representing 7 (**b**). The horizontal lines indicate the mean (±s.e.m.). *P < 0.05, **P < 0.01, determined by Mann One-Way Anova (**b**).

Recently, pre-cancerous colon polyps were shown to be enriched for IL-10 producing NKT cells^43^. We reasoned that IL-10 producing, YY1^lo^ NKT cells might be enriched in tumors. To test this possibility, 5 ×10^5^ CT26 colon cancer cells^44^ were implanted intradermally into BALB/cJ mice. When tumors reached an area between 8 by 8 millimeters and 10 by 10 millimeters (approximately 12–16 days after injection), mice were sacrificed, and hematopoietic cells were isolated from tumors. In contrast to all other studied tissues, nearly 40% of the NKT cells isolated from CT26 tumors were found to be YY1^lo^ (Fig. 7A,B). We next intradermally injected C57BL/6J mice with either 5 ×10^5^ B16F10 melanoma cells^45^ or 5 ×10^5^ D4M3A melanoma cells^46,47^. For both tumors, we once again found that a high percentage (more than 35%) of the tumor infiltrating NKT cells were YY1^lo^ (Fig. 7A,B). Therefore, enrichment for YY1^lo^ NKT cells occurs in different tumor types and in different mouse genetic backgrounds. Efforts to detect IL-10 expression by the tumor infiltrating NKT cells were not, however, successful. It is possible that YY1^lo^ NKT cells infiltrate tumors quickly after implantation and only secrete IL-10 for a short period of time^48^. We attempted, therefore, to isolate leukocytes from tumors at earlier time points (3–5 days post tumor implantation) but were unable to collect sufficient cells for analysis.

## Discussion

Functionally distinct NKT cell effector subpopulations, primed to secrete unique combinations of cytokines and defined by particular transcription factor expression patterns, potently modulate disparate aspects of immunity^49–52^. In this present study, we uncovered a small population of NKT cells that expressed low levels of the transcription factor YY1. YY1^lo^ NKT cells, irrespective of their tissue or mouse strain of origin, shared a distinct transcription factor expression pattern (PLZF^lo^, Tbet^lo^, RORγT^−^, and ThPOK^lo^) as compared to NKT1, NKT2, or NKT17 cells. When activated, YY1^lo^ NKT cells produced little of the typical cytokines, IL-4 and IFN-γ, but instead secreted IL-10. Based upon the unique transcription factor expression pattern and cytokine production capacity of YY1^lo^ NKT cells, we propose that we have identified a novel NKT cell effector subpopulation.

The signals that drive NKT cells to adopt different effector lineages remain incompletely described. One potential mechanism is differential TCRVβ chain usage, as certain NKT cell effector lineages display greater usage of specific TCRVβ chains^39^. However, no substantial TCRVβ usage differences were observed between YY1^lo^ and YY1^hi^ NKT cells. Alternatively, some NKT cell effector lineages, such as the T follicular helper NKT (NKT_fh_) cell, appear to be induced when mature NKT cells are activated under very specific conditions^53^. The YY1^lo^ NKT cell phenotype does not seem to be inducible in mature NKT cells, as the frequency of YY1^lo^ NKT cells was not affected by age, tissue of origin, or *in vivo* activation. Rather, mature YY1^lo^ NKT cells are likely derived from immature, stage 1 YY1^lo^ NKT cells found in the thymus. Why some stage 1 NKT cells express low levels of YY1 remains to be determined. It should be noted that our data do not rule out the possibility that YY1^lo^ NKT cells in the thymus are actually mature cells that have returned. Should this be the case, it may impact the interpretation that YY1^lo^ NKT cells are a distinct lineage.

Consequential differences in YY1 expression levels between helper T cells and Tregs have previously been reported^30^. Knockdown of YY1 in Th2 cells, which usually express high levels of YY1, dramatically reduced their expression of several Th2 cytokines including IL-4, IL-5, and IL-13^31^. Conversely, over expression of YY1 in Tregs, which usually express relatively low levels of YY1, led to their functional impairment and limited their production of IL-10^30^. Experimental evidence from other systems including embryos^54^ and hematopoietic stems cells^26^ provide additional evidence that YY1 dosage is critical for the normal development and function of cells. Our findings, which demonstrate that functionally distinct NKT cell effector populations express different levels of YY1, are therefore consistent with previous studies.

YY1^lo^ NKT cells appear to be a “natural” source of IL-10, as the cells constitutively expressed IL-10 mRNA and rapidly secreted the cytokine following primary activation. Steady state production of IL-10 by YY1^lo^ NKT cells may modulate the general tone of the immune system, serving such functions as maintaining adipose tissue homeostasis. This would be analogous to the constitutive production of IL-4 by NKT cells, which drives the development of “innate-like” CD8 T cell populations^55,56^. Furthermore, the rapid production of IL-10 by YY1^lo^ NKT cells after primary activation may serve a critical function in limiting immune-mediated tissue injury.

YY1^lo^ NKT cells were found at a high frequency in tumors, irrespective of the tumor type or recipient mouse strain. Given the capacity of YY1^lo^ NKT cells found in other tissues to produce IL-10, we hypothesized that YY1^lo^ NKT cells would secrete IL-10 in tumors, but at least at the time point tested, which was ~12 days after tumor implantation, the cells did not express IL-10. It is possible that YY1^lo^ NKT cells infiltrate tumors soon after implantation, and secrete IL-10 for only a brief period. This would be similar to what is found recently found during the immune response to flu, where NKT cell produced IL-4 is made from 3–7 days post infection, but not after day 9^48^. An alternative explanation is that YY1^lo^ NKT cells found in tumors are functionally distinct and unrelated to YY1^lo^ NKT cells found in other tissues.

In accordance with previous studies, we found that a higher frequency of NKT cells secreted IL-10 after secondary activation^21^. Activation, however, does not cause the expansion of the YY1^lo^ NKT cell population, but rather results in an IL-10 producing NKT cell population that expressed high levels of YY1. These induced IL-10 producing NKT cells, therefore, appear to be distinct from the YY1^lo^ NKT cells reported here.

In summary, we found that the ubiquitously expressed transcription factor, YY1, is differentially expressed in NKT cells. Low YY1 expression marks a functionally distinct NKT cell effector lineage that is a natural source of IL-10. It remains to be determined what factors drive the development of this lineage, but we have ruled out differential TCRVβ usage. Furthermore, activation does not lead to the specific expansion of YY1^lo^ NKT cells, but rather induces YY1^hi^ NKT cells to produce IL-10. Our data also suggest that YY1^lo^ NKT cells might impact the tumor microenvironment, but future studies will have to explore this more thoroughly.

## Methods

### Animals

C57Bl/6J mice and BALB/cJ mice were obtained from Jackson Labs. YY1 *flox/flox* mice were obtained from the Jackson Laboratory (Bar Harbor, ME) and, also from a Dr. Michael Verzi (Rutgers University). PLZF-Cre mice were developed in our lab as reported^14^. IL-10 GFP mice were obtained from Jackson Laboratories. All mouse strains were bred and maintained in the animal facility of the Child Health Institute of New Jersey. All experimental protocols and procedures were approved by the Institutional Animal Care and Use Committee of the Child Health Institute of New Jersey. Animal care and experimental procedures were carried out in accordance with the guidelines of the Institutional Animal Care and Use Committee of Rutgers University and the National Institutes of Health Guide for the Care and Use of Laboratory Animals.

### Tumor cell lines

The CT26 and B16F10 cell lines were obtained from Dr. Andrew Zloza (Rush University). The D4M3A cells were provided by Dr. David Mullins (Dartmouth University). Cells were cultured as monolayers at 37 degrees Celsius and 5% CO_2_ in RPMI (CT26 and D4M3A) or DMEM (B16F10) supplemented with 10% heat-inactivated bovine serum and 0.5% penicillin G-streptomycin sulfate (Corning). 0.25 trypsin EDTA (Corning) was used to detach adherent cells.

### Tumor implantation into mice

5×10^5^ CT26 colon cancer cells were injected intradermally into BALB/c mice. 5×10^5^ B16F10 melanoma cancer cells or D4M3A melanoma cancer cells were injected intradermally into C57Bl/6 mice. Tumor growth was monitored daily. Mice were sacrificed and tumors were harvested when they reached an area between 8×8 millimeters and 10×10 millimeters.

### Preparation of single cell suspensions from lymphoid and non-lymphoid tissues

Thymus, spleen, liver, and lung were harvested and dissociated between glass slides and filtered through a 40-μm mesh. Spleen and thymus samples were treated with Red Blood Cell Lysing Buffer (Sigma) and counted on a hemocytometer. Liver homogenate was suspended in Percoll (Sigma) to a final concentration of 30% Percoll. Liver homogenates were then centrifuged for 20 min at 500 r.c.f. in a Sorvall RTH-750 rotor. Lymphocytes were then collected from the buffy layer, washed once with FACS buffer, treated with Red Blood Cell Lysing Buffer (Sigma) and counted on a hemocytometer. Lung homogenate was underlayed with lympocyte seperation medium (MP Biomedicals) and then centrifuged for 20 min at 500 r.c.f. in a Sorvall RTH-750 rotor. Lymphocytes were then collected from the buffy layer, washed once with FACS buffer, and counted on a hemocytometer. Visceral adipose tissue was harvested from mice and minced. Minced adipose tissue was suspended in RPMI medium containing collagenase Type IV (Worthington) at a concentration of 1 mg/ML and rotated at 37 degrees Celsius for 45 minutes. After rotation, cells were washed with RPMI. Tumors were collected from mice when they reached an area between 8×8 millimeters and 10×10 millimeters. Tumors were suspended in RPMI medium containing collagenase Type IV (Worthington) at a concentration of 1 mg/ML. Tumors were dissociated using gentleMACs (Miltenyi Biotec) program m_impTumor_01_01. Dissociated tumors were incubated with rotation for 45 minutes at 37 degrees Celsius and washed with FACs buffer and filtered through 40-μm filters.

### Flow cytometry, antibodies, automacs enrichment, and cell sorting

Single cell suspensions were blocked in FACS buffer with 2% normal mouse serum, 0.1% anti-Fcγ antibody, and 0.1ug/ml streptavidin for 15 minutes at 4 C followed by staining with primary antibodies for 20 minutes at 4 C. Intracellular staining for transcription factors and cytokines was accomplished using the Foxp3/Transcription Factor Staining Buffer Set (eBioscience). All intracellular staining was performed at room temperature. IL-10 was detected using the Mouse IL-10 Secretion Assay - Detection Kit (Miltenyi Biotech). The following antibodies were used in this study: anti-CD3 (500A2), Anti-CD4 (RM4-5), anti-CD8α (53–6.7), anti–IL-17A (Ebio17B7), anti-CD19 (1D3), anti-CD24 (M1/69), anti-CD44 (IM7), CD45.2 (104), anti-CD69 (H1.2F3), anti-TCRVβ 8.2 (MR5-2), anti-TCRVβ7 (TR310), anti-TCRVβ2 (B20.6), anti-IFN-γ(XMG1.2), anti-IL4 (11B11), anti-MHCII (212.A1), PD-1 (J43), ICOS (15F9), CD49d (R1-2), NRP1 (3DS304M), anti-NK1.1 (PK136), anti-PLZF (Mags.21F7), anti-RORγT (B2D), anti-T-bet (eBio4B10), anti-YY1 (sc-7341), anti-ThPOK (2POC), FoxP3 (FJK-16s). The PBS57-loaded CD1d-tetramer was provided by the National Institutes of Health tetramer core facility. Dead cells were excluded when possible, by DAPI staining and doublet events were eliminated by comparing FSC-W to FSC-H and SSC-W to SSC-H. Events were acquired on a LSRII cytometer (BD Biosciences, San Jose, CA), and the data were analyzed with the FlowJo software (TreeStar, Ashland, OR).

NKT cell enrichment was performed using an AutoMACS (Miltenyi Biotec). Cells were stained with the PE-labeled CD1d tetramer, incubated with anti-PE microbeads (Miltenyi Biotec), and separated on the AutoMacs (Miltenyi Biotec). Samples were at least 80% pure following enrichment.

Cell sorting was performed at the Flow Cytometry/Cell Sorting & Confocal Microscopy Core Facility at Rutgers EOSHI or at the Cancer Institute of NJ.

### *In vivo* and *In vitro* activations

Mice were injected intravenously via the retro orbital route with 10 ug of the NKT cell specific agonist α-galactosylceramide (α-GalCer). 90 minutes after injection, splenocytes were harvested.

Following enrichment by Automacs (Miltenyi Biotech) NKT cells were activated *in vitro* by incubation with phorbol 12-myristate 13-acetate (50 ng/ml) and ionomycin (500 ng/ml) for 3 hours or 5 hours when detecting IL-10 or IL-4/IFN-γ respectively. The Mouse IL-10 Secretion Assay - Detection Kit (Miltenyi Biotech) was then utilized to assess the production of IL-10 by NKT cells.

### RNA isolation and quantitative PCR analysis

Sorted NKT cells were lysed in Trizol solution at a concentration of 1×10^6^ cells per mL. RNA was isolated from the trizol solution using the Direct-zol RNA MicroPrep Kit(Zymo Research). Reverse transcription was performed using the GoScript Reverse Transcription System (Promega). The resultant cDNA was utilized to perform TaqMan (Life Technologies) based qPCR. Taqman Universal PCR Master Mix No AmpErase UNG (Life Technologies) was used. The following TaqMan probes were used: B2M (Mm00437762_m1) and IL-10 (Mm01288386) Samples were run on a QuantStudio6 Flex Real Time PCR System(Life Technology). Relative expression of IL-10 was calculated by using the ΔΔCT method, using B2M as the internal housekeeping control gene.

### General experiment design and statistical analysis

Data from at least three samples in three or more independent tests were collected as detailed in the figure legends. Statistical analysis was performed using Microsoft Excel or GraphPad Prism. All data was subjected to analysis with Mann Whitney U-Test or One-Way Anova.

## Supplementary information

Supplementary Information.
