# Supplementary material for: YY1lo NKT cells are dedicated IL-10 producers
Source: Sci Rep. 2020 Mar 3;10:3897. doi: 10.1038/s41598-020-60229-6 (PMC7054430; doi:10.1038/s41598-020-60229-6)
Supplement: Supplementary file 1 — Supplementary Information. [file 41598_2020_60229_MOESM1_ESM.pdf]

YY1^{lo} NKT cells are dedicated IL-10 producers

Patrick W. Darcy[3], Lisa K. Denzin[1,2,3] and Derek B. Sant'Angelo[1,2,3]*

[1]Graduate School of Biomedical Sciences
[2]Department of Pediatrics
[3]Child Health Institute of New Jersey
Rutgers Robert Wood Johnson Medical School
New Brunswick, NJ 08901, USA

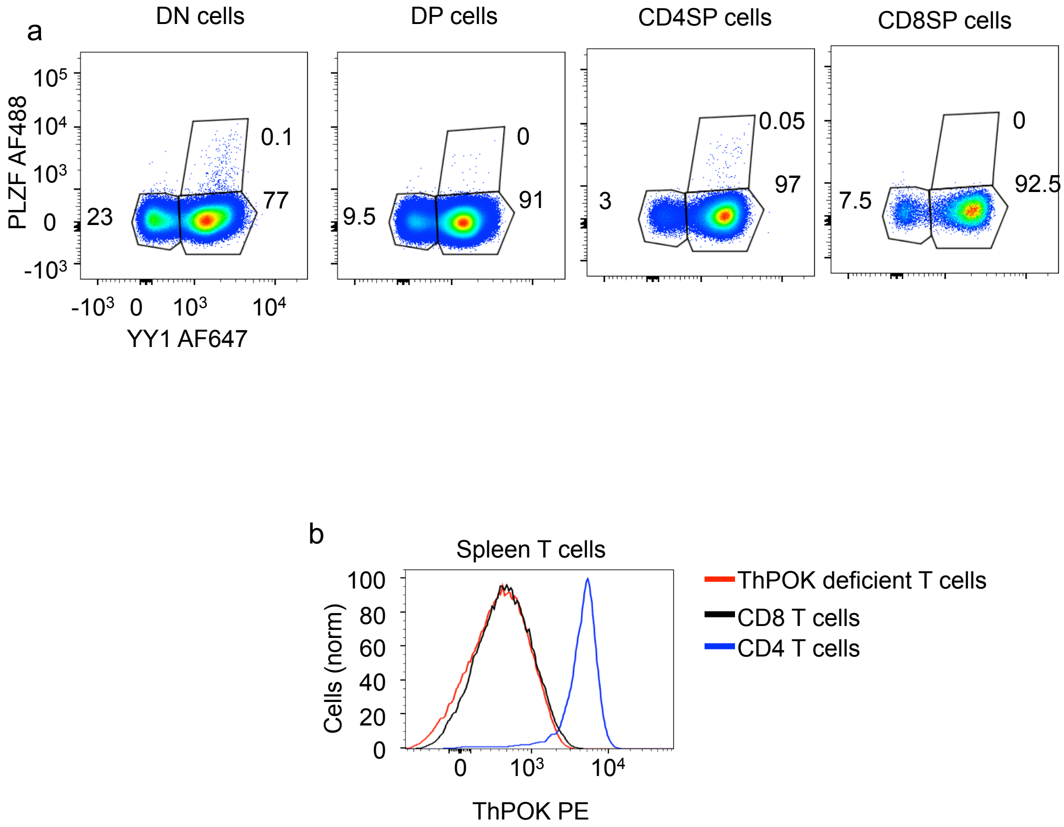

Figure S1. YY1 expression in thymocytes and ThPOK Staining Control (Related to Figure 1 and Figure 3)
(A) DN (CD4- CD8-), DP (CD4+, CD8+), CD4SP (CD4+, CD8-), and CD8SP (CD4-, CD8+) thymocytes from C57BL/6 mice were stained for YY1 and PLZF. (B)T cells from C57BL/6 or ThPOK KO mice spleens were stained for ThPOK. ThPOK deficient T cells and CD8 T cells do not express ThPOK, as compared to wild type CD4 T cells.

Supplementary
Figure 1

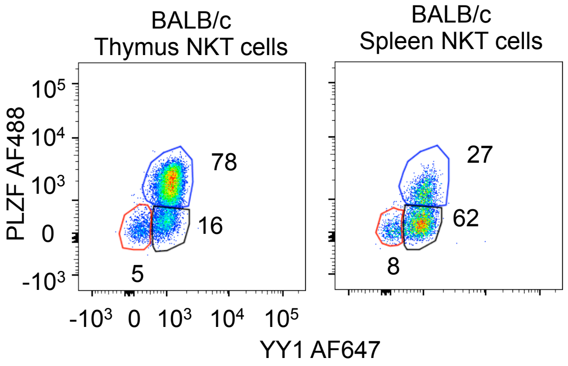

Figure S2. YY1^{lo} NKT cells in BALB/c mice. (Related to Figure 3)
Leukocytes were isolated from the spleen and thymus of BALB/c mice and analyzed by FACs. Representative FACs plots of YY1 and PLZF expression in NKT cells (MHCII-, CD3+, CD1dtet+, CD24-) are shown. N=5.

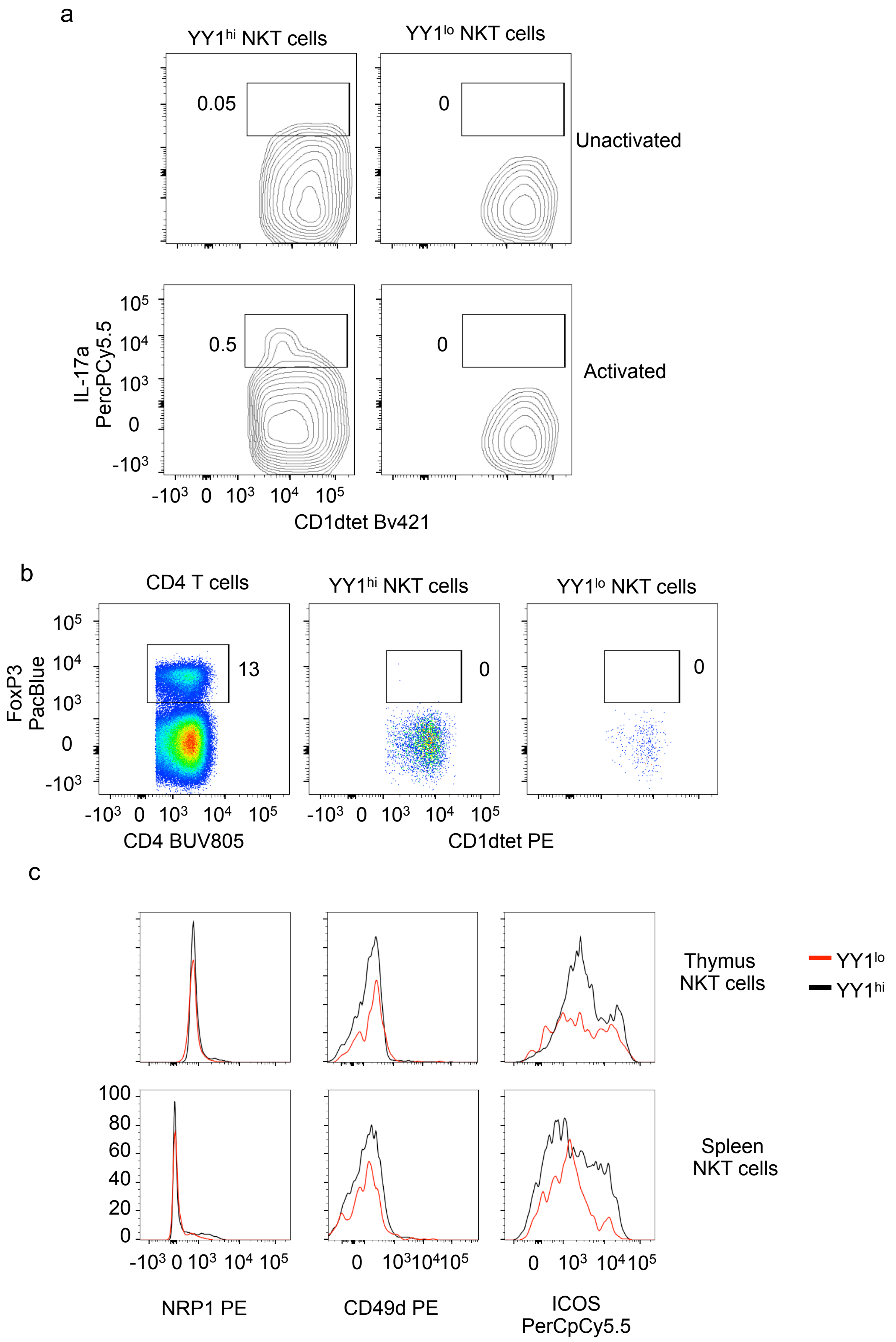

Figure S3. YY1^{lo} NKT cells cytokine, transcription factor, and cell surface phenotype. (Related to Figures 4 and 6) (A) Spleen NKT cells were isolated from C57BL/6 mice 90 minutes after injection of α-GalCer and stained for the cytokine IL-17a. (B) Thymus CD4SP T cells and NKT cells were stained for FoxP3. (C) Thymus and Spleen NKT cells were stained for NRP1, CD49d, and ICOS. Representative FACs plots are shown. N=5.

Supplementary Figure 3

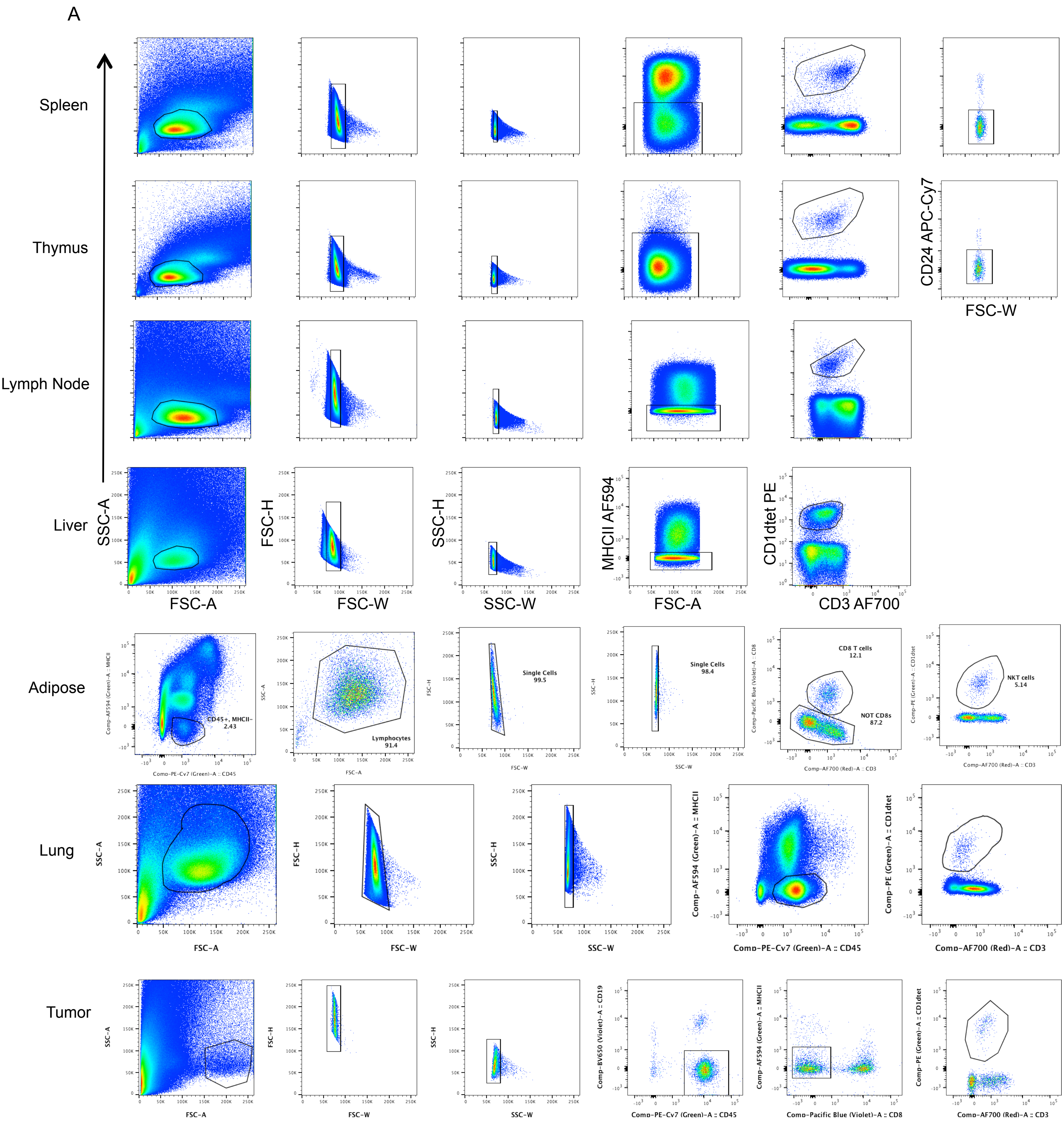

Figure S4. Gating Schemes (Related to Figures 1-7)
Gating schemes used to identify the cells of interest in the main text figures are shown above.
